# *Methanobrevibacter oralis*: a comprehensive review

**DOI:** 10.1080/20002297.2024.2415734

**Published:** 2024-11-01

**Authors:** Virginie Pilliol, Boualam Mahmoud Abdelwadoud, Hamiech Aïcha, Tellissi Lucille, Aboudharam Gérard, Tassery Hervé, Drancourt Michel, Grine Ghiles, Terrer Elodie

**Affiliations:** a Aix-Marseille Université, Microbes Evolution, Phylogénie et Infection (MEPHI), Marseille, France; b Aix Marseille Université, Assistance Publique des Hôpitaux de Marseille (Ecole de Médecine Dentaire), Microbes Evolution, Phylogénie et Infection (MEPHI), Marseille, France; c Institut Hospitalo-Universitaire (IHU) Méditerranée Infection, Marseille, France

**Keywords:** Methanogen, oral microbiota, dysbiosis, periodontitis, endodontic infection, abscess, ancient dental calculus

## Abstract

*Methanobrevibacter oralis* (*M. oralis*) has predominated human oral microbiota methanogenic archaea as far back as the Palaeolithic era in Neanderthal populations and gained dominance from the 18^th^ century onwards. *M. oralis* was initially isolated from dental plaque samples collected from two apparently healthy individuals allowing its first characterization. The culture of *M. oralis* is fastidious and has been the subject of several studies to improve its laboratory growth. Various PCR methods are used to identify *M. oralis*, targeting either the 16S rRNA gene or the *mcrA* gene. However, only one RTQ-PCR system, based on a chaperonin gene, offers specificity, and allows for microbial load quantification. Next-generation sequencing contributed five draft genomes, each approximately 2.08 Mb (±0.052 Mb) with a 27.82 (±0.104) average GC%, and two ancient metagenomic assembled genomes. *M. oralis* was then detected in various oral cavity sites in healthy individuals and those diagnosed with oral pathologies, notably periodontal diseases, and endodontic infections. Transmission pathways, possibly involving maternal milk and breastfeeding, remain to be clarified. *M. oralis* was further detected in brain abscesses and respiratory tract samples, bringing its clinical significance into question. This review summarizes the current knowledge about *M. oralis*, emphasizing its prevalence, associations with dysbiosis and pathologies in oral and extra-oral situations, and symbiotic relationships, with the aim of paving the way for further investigations.

## Key Points

- *Methanobrevibacter oralis*, the most predominant methanogen in human oral microbiota, traces back to the Palaeolithic era and emerges as the dominant methanogen from the 18th century onwards.
- Our understanding of *Methanobrevibacter oralis* microbiology remains limited, particularly regarding its phenotypic, genomic, and metabolic characteristics. Furthermore, specific identification and quantification methods are still limited.
- Although *Methanobrevibacter oralis* has been found in dysbiotic conditions, such as periodontitis, and in other oral and extra-oral pathologies, its pathogenicity remains largely understudied and should be the focus of future research.

## Introduction

*Methanobrevibacter oralis* (*M. oralis*) is one of ten methanogenic archaea (methanogens) identified in the oral microbiota [1], alongside *Methanobrevibacter smithii* (*M. smithii*) and *Methanobrevibacter massiliense* (*M. massiliense*), all three of which were isolated through culture methods [2]. *M. oralis* was originally isolated in 1994 from two dental plaque samples collected from two apparently healthy individuals [3]. Since then it has consistently emerged as the most prevalent methanogen associated with the oral microbiota [4], exhibiting a dynamic relationship with human evolution, dating back to the Palaeolithic era in Neanderthal populations [5–8].

Exploration of *M. oralis* surpasses mere confirmation of its presence. The understanding of whether *M. oralis* acts as a pathogen or an opportunist remains uncertain. However, the quantification of *M. oralis* has emerged as a potential diagnostic biomarker and a therapeutic target for some oral diseases [9], given its distinctive antibiotic resistance profile [10]. Its intricate interactions within the oral microbiota, involving various microorganisms such as bacteria [11] and nanoarchaea [12], reveal a complex network of relationships particularly important in the context of dysbioses. Unravelling these complex interactions and dependencies within microbial ecosystems could provide valuable insights into the role of *M. oralis* in maintaining microbial balance and influencing health outcomes. Beyond its oral domain, *M. oralis* extends to an extra-oral realm, with notable occurrences in brain abscesses [13,14]. This extra-oral presence suggests a potential for systemic implications and prompts a reassessment of its medical significance.

This review aims to provide an overview of current knowledge about *M. oralis*, including its culture and detection methods, and focus on its prevalence within the human microbiota, particularly in the context of oral dysbiosis or abscess-related pathologies. Additionally, we examine its associations with other microorganisms and discuss its potential dual role as both a commensal microorganism and a pathogen. By shedding light on the various aspects of *M. oralis* microbiology, this review may pave the way for further investigations into the intricate dynamics of the human microbiota, particularly in the context of oral dysbiosis.

## Antiquity of *M. oralis*

DNA Illumina sequencing firstly detected the presence of *M. oralis* in two middle Palaeolithic (about 50 000 BP) sediment samples from the El Salt site in Spain, containing millimetric coprolites and faecal biomarkers in sufficient proportions to suggest a *Homo* origin [7]. However, the oldest and clearer evidence for *M. oralis* in human oral microbiota was obtained by metagenomic analysis of a dental calculus sample collected from an approximately 48 000-year-old Neanderthal individual suffering from a dental abscess, found in El Sidrón cave (sample El Sidrón 1) in Spain [5]. Indeed, this sample yielded an almost complete *M. oralis* genome sequence named *M. oralis* subsp. *neanderthalensis*, thought to have diverged from the modern *M. oralis* strain JMR01 about 12 600 years BP. This divergence seems to have occurred far later than the genomic divergence of Neanderthals from *Homo sapiens,* which took place between 45̵ 000 to 75 000 years BP, suggesting that *M. oralis* strains likely differed between Neanderthals and modern humans, leading to the emergence of *M. oralis* subsp. *neanderthalensis* [5]. In a subsequent study, a taxonomic analysis was performed on previously published data (including those from [5] using a nucleotide-to-nucleotide alignment with MALT (MEGAN Alignment Tool) against an extended database (RefSeqGCS, https://doi.org/10.25909/5b84ddf58ac49) [6]. This analysis revealed the presence of *M. oralis* in an additional Neanderthal dental calculus sample from the Spy Cave (Spy II) in Belgium, dated to 36,000 years BP [6].

**Figure 1.**
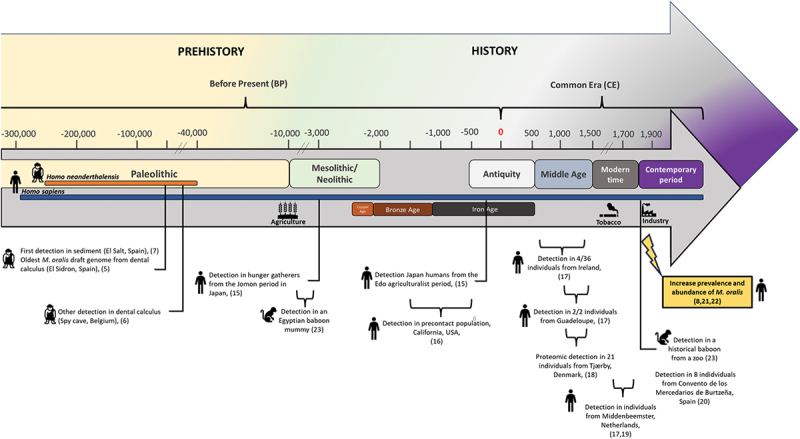
Timeline of *M. oralis* antiquity. This figure provides a chronological timeline highlighting the periods and locations pertinent to *M. oralis* antiquity. It illustrates key milestones and their correlation with significant historical and prehistorical periods, emphasizing their relevance to the study of *M. oralis*.

Later in the timeline illustrated in Figure 1, *M. oralis* was also detected in ancient Japanese *Homo sapiens*, spanning both the ‘Jomon’ hunter-gatherer period (3000 years BP) and the ‘Edo’’ agriculturalist period (400–150 years BP) by dental calculus aDNA metagenomics [15]. Additionally, this study highlighted a higher abundance of *M. oralis* in women with periodontal disease (32%), evidenced by higher levels of bone loss during the agriculturalist ‘Edo’ period than in men (5%) but without statistical difference, only 10 individuals were included. Likewise, *M. oralis* was reported in dental calculus samples collected from nine individuals (dating from 1479 to 495 years BP) in California in the United States [16].

In a broad metagenomic investigation spanning various historical sites, *M. oralis* was identified exclusively in dental calculus in seven individuals: 4/36 (11%) from Ireland (600–1300 Common Era (CE), 2/2 (100%) from Guadeloupe (975–1395 CE), and 1/2 (50%) from the Netherlands (1611–1866 CE) [17]. Metaproteomics analysis of dental calculus sampled from 21 individuals buried in a 1100–1450 CE mediaeval cemetery in Tjærby, Denmark, detected several proteins from *M. oralis* which were found to be significantly more abundant in the group with an abundance of periopathogenic species compared to the other group associated with oral health [18]. *M. oralis* was also found in higher abundance in 76 individuals from Middenbeemster in the Netherlands dated from 1611–1866 CE than in the 31 individuals from various other sites [19]. This was confirmed by another metagenomics study including 65 individuals from the same site of Middenbeemster and eight from Convento de los Mercedarios de Burtzeña (CMB), Spain, both dated from the 19^th^ century industrial era [20].

PCR-sequencing of hundreds of dental calculus samples collected from six archaeological sites in France, dated from the 14^th^ to the 19^th^ century detected *M. oralis* in 11/56 (19.6%) of samples free of PCR inhibition (56/100, 56%), with significantly lower prevalence in past populations compared to modern ones [21]. Additionally, the metagenomic reconstruction of oral microbiomes from 44 ancient foragers and farmers in the Balkans and the Italian peninsula spanning a large period from the Palaeolithic era to the Early Middle Ages, and comparison with historical samples confirmed the increased abundance of *M. oralis* in historical samples from the 18th and 19^th^ centuries [22]. Furthermore, a comprehensive analysis spanning the Neolithic period to the contemporary period, focusing on methanogen diversity and evolution in the oral microbiome, revealed two previously unidentified archaeal species which were predominant before the 18^th^ century [8]. Intriguingly in this study, *M. oralis* emerged in samples from the Middle Ages, and was not detected in older samples and became the dominant methanogen from the 18^th^ century onwards, while the other species declined [8].

In a non-human study exploring the oral microbiome of ancient Egyptian baboons from the end of the pharaonic era (9^th^–6^th^ centuries BP) and historical baboons from the 19^th^ century through metagenomic analysis, *M. oralis* was identified in one historical baboon and one Egyptian mummy. This presence is likely to be attributable to horizontal foodborne transmission, a consequence of captive breeding practices [23].

In essence, *M. oralis* appears not to be historically confined to *H. sapiens*, as it has been found in Neanderthals. Indeed, the earliest evidence of *M. oralis* was identified in coprolitic sediment [7] and dental calculus [5] from these hominids. Neanderthal or *Homo neanderthalensis* represented a species closely related to *Homo sapiens*. Inhabiting Europe and Western Asia between approximately 400 000 and 40 000 years ago, both species displayed distinctive anatomical features, including robust bodies and elongated skulls [24]. Neanderthals exhibited adaptations to diverse environments. Proficient hunters and gatherers, they adhered to an omnivorous diet, using a range of resources from large animal meats to plant foraging [5,24]. The draft genome of *M. oralis neanderthalensis* was recovered from a Neanderthal at the El Sidrón site in Spain associated with a non-meat diet [5]. However, its subsequent detection at the meat-eating Spy site in Belgium contradicted the hypothesis that diet influenced its presence in Neanderthals [6]. *M. oralis* was further detected in prehistoric *H. sapiens,* and possible transmission between the two *Homo* species is still open [5]. The advent of agriculture at the beginning of the Neolithic area appears to have led to an increased abundance of *M. oralis* [15]. Nonetheless, the precise impact of agriculture on oral microbiota remains uncertain, as the modifications appeared to have unfolded gradually [22]. Interestingly, the introduction of tobacco in Europe during the 16^th^ century did not seem to have a discernible impact on *M. oralis* [20]. Later, *M. oralis* was sporadically detected in numerous studies and identified in populations across several continents, including the pre-contact populations in America [16]. This underscores the widespread presence of *M. oralis* in *H. sapiens* long before the onset of the European colonisation of America [16]. A notable shift in its abundance and prevalence occurred in Europe [8,21,22]. Collectively, these three studies suggest a notable rise in the prevalence and abundance of *M. oralis*, particularly from the 18^th^ century onwards, possibly influenced by societal changes and potentially linked to the generalisation of sugar consumption during the industrial era. This raises questions about changes in bacterial composition that may favour micro-environments which are conducive to *M. oralis*. Looking for bacteria associated with *M. oralis*, which can degrade sugars, could provide valuable insights for future investigations, and may explain why *M. oralis* became the predominant oral methanogen. Further investigations into the evolutionary timeline of *M. oralis* across different human populations in different parts of the world and its potential interactions with dietary and lifestyle changes could provide deeper insights into its prevalence and significance in oral microbiomes over time.

## General microbiology

### First insights into oral methanogens and the isolation of *M. oralis*

The search for methanogenic archaea in the human oral cavity began in 1987 by culturing samples of dental plaque from patients who had not brushed their teeth for 24 h [25]. The authors aimed to find methanogens in the subgingival plaque from the gingival crevice, an ecological niche favourable to the growth of anaerobic microorganisms [25]. Methanogens belonging to the *Methanobrevibacter* genus were isolated from three of ten samples, confirming their presence in the oral cavity [25]. Along with this first study, another study used the culture approach to reveal the presence of methanogens in the dental plaque of patients with periodontal disease [26]. The predominantly cultivated genus was *Methanobrevibacter*. Finally, in 1994, *M. oralis* was isolated and characterised from the subgingival plaque of two apparently healthy patients, and the results were published by Ferrari et al. (strain DSM 7256) [3].

### Phenotypic characterisation

**Figure 2.**
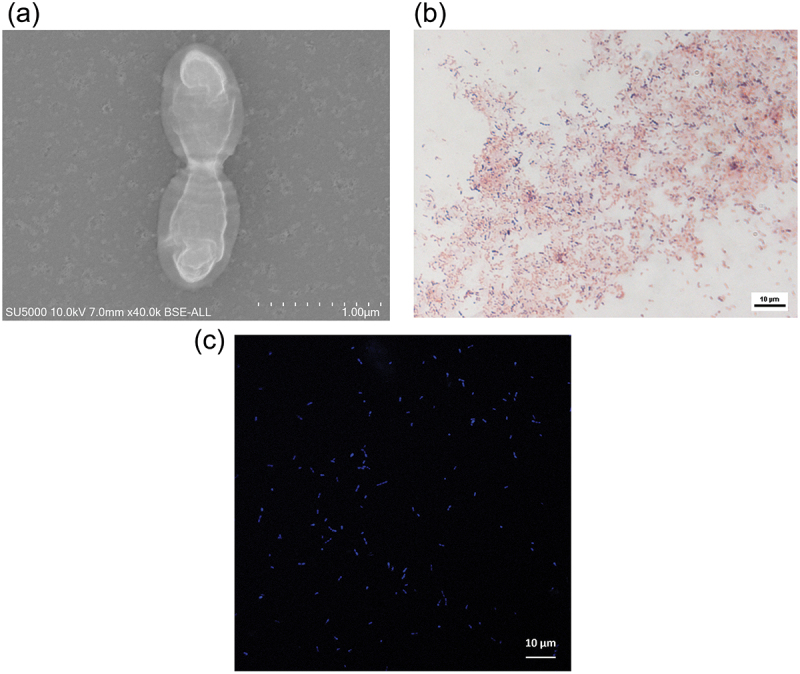
Microscopy features of *M. oralis* DSM 7256. (a). Electron microscopy: high-resolution electron microscopy (SU5000 hITACHI, 10 KV, X 40,000) reveals *M. oralis* diplococcobacilli with distinct external cell walls and internal membrane. (b). Gram staining: *M. oralis* is observed as gram-variable coccobacilli, appearing both gram-positive and gram-negative, typically arranged in pairs or short chains. (c). Confocal microscopy: visualization of *M. oralis* under confocal microscopy (LSM 900, Carl Zeiss microscopy GmbH) shows autofluorescent coccobacilli and diplococcobacilli emitting blue fluorescence at 420 nm.

*M. oralis* has been described as a non-motile, non-spore-forming coccobacillus with tapered ends or short oval rods of 0.4–0.5 µm in width and 0.7–1.2 µm in length (Figure 2a), observed by two or short chains. *M. oralis* is gram-positive to gram-variable after four days of culture (Figure 2b) and is autofluorescent at 420 nm, as are other methanogens (Figure 2c). Less is known about its cell wall and membrane, contrary to other methanogens, but in transmission electron microscopy, *M. oralis* harbours a tri-stratified wall with deep invaginations [3].

### Molecular detection and identification

Molecular detection is typically carried out using PCR and RT-PCR, sometimes supplemented by sequencing for identification (Table 1). These methods target the 16S rRNA gene or the *mcrA* gene involved in methanogenesis. Five systems targeting the 16S rRNA gene and three systems targeting the *mcrA* gene have been developed to detect Archaea or methanogens, including *M. oralis*. Only one *M. oralis*-specific RT-PCR system targeting a chaperonin gene allows for the quantification of *M. oralis* [9]. Moreover, three Fluorescent In Situ Hybridization (FISH) probes have been designed to detect archaea, including *M. oralis*, also targeting the 16S rRNA gene or the *mcrA* gene. Additionally, metagenomics and Next-Generation Sequencing (NGS) have detected *M. oralis* in various samples [48,49], including ancient specimens [5–8,15,17,19,22,23,50], although a large study of over 1000 gut samples detected *M. oralis* in only one sample [51].

**Table 1.** The different PCR systems able to detect and amplify *M. oralis* sequences.

Primer pair	Sequences (5’ − 3’)	Target DNA	Amplicon size (approximative)	Temperature profile	Molecular analysis	Reference
300fEyAr	AGC(A/G)(A/G)GAGCCCGGAGATGG	16S rRNA	650	95°C (1 min), 35 cycles of 95°C (15s), 64°C (30s), 72°C (15s), and 72°C (7 min)	Standard PCR	Kulik et al., 2001 [27]; Faveri et al., 2011 [28]; Prakash et al., 2020 [29]
954rEyAr	CGGCGTTGA(A/G)TCCAATTAAAC					
SDArch0333aS15	TCCAGGCCCTACGGG	16S rRNA	530	35 cycles of 94°C (30s), 58°C (30s), and 72°C (30s), and 72°C (3 min)	Standard PCR	Lepp et al., 2004 [4]; Vickerman et al., 2007 [30]; Yamabe et al, 2008 [31]; Mansfield et al., 2012 [32]; Drancourt et al., 2017 [13]; Nkamga et al., 2018 [14]; Grine et al., 2018 [33]; Togo et al., 2019 [34]; Sogodogo et al., 2019 [35]; Guindo et al., 2020 [36]; Hassani et al., 2020 [37], 2021 [12]; Djemai et al., 2021 [38], 2022 [39]
S*Univ0515aA19	(FAM-)TTACCGCGGCKGCTGGGACTAMRA			95°C (10 min), 50 cycles of 95°C (30s), 55°C (30s), 60°C (45s), 65°C (15s), and 72°C (15s)	ABI Prism 7900HT Sequence Detection System (Applied Biosystems)	Lepp et al., 2004 [4]
A109F	ACKGCTCAGTAACACGT	16 r RNA	798	95 °C (10 min); 40 cycles of 95 °C (10s), 65 °C (10s), and 72 °C (45s)	Standard PCR, Sybergreen RTQ-PCR and sequencing	Horz et al., 2015 [40]; Brzezińska-Błaszczyk et al., 2018 [41]; Vianna et al., 2006 [42], 2008 [43], 2009 [44]
A934R	GTGCTCCCCCGCCAATTCCT					
Mbb279F	TGATCGGTACGGGTTGTG	16Sr RNA	405	95°C (10 min); 35 cycles of 95°C (10 s), 58°C (10 s) and 72°C (25 s); fluorescence measurement at 78° C	Sybergreen RTQ-PCR and sequencing	Horz et al., 2015 [40]
Mbb709R	CAACAGGCGGTCCTCCCA					
Metha_16S_2_MBF	CGAACCGGATTAGATACCCG	16Sr RNA	/	50°C (2 min), 39 cycles of 95°C (5 min), 95°C (5s), and 60°C (30s)	RT-PCR	Drancourt et al., 2021 [45]; Guindo et al., 2020 [36]; Djemai et al., 2021 [38], 2022 [39]
Metha_16S_2_MBR	CCCGCCAATTCCTTTAAGTT					
FAM_Metha_16S_2_MBP	FAM- CCTGGGAAGTACGGTCGCAAG					
ME1	GCMATGCARATHGGWATGTC	mcrA	760	30 cycles of 94°C (40s), 50°C (1 min and 30 s), 72°C (3 min), and 72°C (10 min)	Standard PCR	Scalan et al., 2008 [46]
ME2	TCATKGCRTAGTTDGGRTAGT					
LuF	GGTGGTGTMGGATTCACACART AYGCWACAGC	mcrA	470	95 ° C (10 min); 40 cycles of 95 ° C (10s), 56 ° C (7s), and 72 ° C (25s)	Sybergreen RTQ-PCR and sequencing	Vianna et al., 2006 [42], 2008 [43] and 2009 [44]; Horz et al., 2012 (108); Huynh et al., 2015 [2], 2016 [21]; Nkamga et al., 2018 [14]; Belkacemi et al., 2018 [47]; Grine et al., 2018 [33]
LuR	TTCATTGCRTAGTTWGGRTAGTT					
mcrAFor	GCTCTACGACCAGATMTGGCTTGG	mcrA	/	35 cycles of 94°C (30s), 58°C (30s), and 72°C (30s), and 72°C (3 min)	Standard PCR	Sogodogo et al., 2019 [35]
mcrARev	CCGTAGTACGTGAAGTCATCCAGCA					
*M. oralis*-cnp602F	GCTGGTGTAATCGAAC CTAAACG	Chaperonin gene cnp60	/	95°C (5 min), 40 cycles of 95°C (1s), 60°C (35s), and 45°C (30s)	RTQ-PCR	Bringuier et al., 2013 [9]; Huynh et al., 2015 [2], 2016 [21]; Drancourt et al., 2017 [13]; Togo et al., 2019 [34]; Guindo et al., 2020 [36]; Hassani et al., 2020 [37], 2021 [12]
*M.oralis*-cnp602R	CACCCATACCCGG ATCCATA					
*M.oralis*-cnp602P	FAM-AGCAGTGCACCTGCTGATA TGGAAGG					
Arch 915	GTGCTCCCCCGCCAATTCCT	16S rRNA	N/A	65°C (10 min), and 37°C (20 h)	FISH	Huynh et al., 2016 [21]; Grine et al., 2018 [33]; Sogodogo et al., 2019 [35]; Hassani et al., 2020 [37], 2021 [12]
SBGA-1	Not available	16S rRNA	N/A	65°C (8 h)	FISH	Lepp et al., 2004 [4]
LuR	TTCATTGCRTAGTTWGGRTAGTT	mcrA	N/A	80°C (5 min), and 46°C (16 h)	FISH	Sogodogo et al., 2019 [35]

### Genomes and diversity

Only five draft *M. oralis* genomes were available in the NCBI database at the time of this review (December 2023) (Table 2). The first draft genome, published in 2014, was obtained from the *M. oralis* strain JMR01 isolated in our laboratory from the human gut [52]. A second draft genome of *M. oralis* DSM 7256, isolated from human subgingival plaque in 1994 by Ferrari et al., was published in 2016 [53,54]. Two other draft genomes were deposited by our laboratory, *M. oralis* CSUR P5920 (name M2), isolated from human breast milk [34] and the reference genome of *M. oralis* YH, isolated from dental plaque, along with its nanoarchaeal symbiont, *Nanopusillus massiliensis* [12]. Following these reports, the *M. oralis* genome is about 2.08 Mb (±0.052) with an average 27.82 GC% (±0.104) and an average of 1896 protein coding genes (±41) (Table 2). It harbours two or three CRISPR loci and associated proteins (Cas). Based on the *M. oralis* strain JMR01 draft genome, multispacer sequence typing (MST) using four spacer primer systems revealed at least nine genotypes in *M. oralis*, several variants of which could be carried by a single individual [55].

**Table 2.** *M. oralis* genomes features.

Organism Name	*Methanobrevibacter oralis*	*Methanobrevibacter oralis*	*Methanobrevibacter oralis*	*Methanobrevibacter oralis*	*Methanobrevibacter oralis*	*Methanobrevibacter oralis*	*Methanobrevibacter oralis*
Organism Groups	Archaea;Euryarchaeota	Archaea;Euryarchaeota	Archaea;Euryarchaeota	Archaea;Euryarchaeota	Archaea;Euryarchaeota	Archaea;Euryarchaeota	Archaea;Euryarchaeota
Strain	YH	DSM 7256	MGYG-HGUT-02162	M2 CSUR P5920	JMR01	N/A	N/A
BioSample	SAMEA9459756	SAMN04867341	SAMEA5851666	SAMEA104570764	SAMEA3138857	N/A	N/A
BioProject	PRJEB46774	PRJNA318760	PRJEB33885	PRJEB24872	PRJEB4880	PRJEB43389	N/A
Sample	Human dental plaque	Human dental plaque	Human feces	Human milk	Human feces	Ancient dental calculus from an Early Middle Age *Homo sapiens*	Ancient dental calculus from a Neanderthal
Assembly	GCA_912073625.1	GCA_001639275.1	GCA_902384065.1	GCA_900289035.1	GCA_000529525.1	N/A	N/A
Total Length (bp)	1,953,936	2,140,433	2,083,511	2,124,480	2,107,831	1,372,986	2,076,642
No. of Sequences	14	136	60	106	14	265	14
GC Content (%)	27.9	27.7	27.8	27.7	27.8	27.6	29.9
N50	308.900	45.270	88.069	45.266	36.2296	5.712	360.780
Gap Ratio (%)	0	0	0.00000096	0.00028713	0.01153888	0	68.406398
No. of CDSs	1.920	2.011	2.279	2.000	2.283	1.153	190
No. of rRNA	2	2	2	2	2	0	2
No. of tRNA	31	30	31	30	31	12	30
No. of CRISPRS	3	2	2	3	2	1	0
Coding Ratio (%)	84.9	80.7	82.1	81.8	81.2	69.5	4.7
Completeness(%)	100	100	95.33	100	95.33	52.41	24.3
Contamination(%)	0	0	0	0	0	0.028	0

### Culture methods

Methanogens are fastidious, they are strictly anaerobic and thus require specific conditions of culture and isolation. The first ever *M. oralis* isolate was cultivated in a modified Balch et al. medium 1, called anaerobic growth medium (MB), a liquid medium preserved in a serum bottle incubated under 80% H_2_/20% CO_2_ atmosphere (202.6 kPa) for eight days [3]. The headspace was repressurised every three to four days [3]. Serial dilutions were then subcultured on an MB agar plate for 15–20 days and multiple further transfers with antibiotics made pure isolation of the *M. oralis* strain DSM 7256 possible. Ferrari et al. detailed the optimal growth conditions according to neutral pH value (6.9 to 7.4), NaCl concentration (0.01 to 0.1 M), temperature (36°C to 38°C), and emphasised the need for a 80% H_2_ /20% CO_2_ atmosphere and the presence of a mixture of volatile fatty acids and faecal extracts. Meanwhile, formate, acetate and methanol were reported as being dispensable [3]. Other studies used the medium 119a [56] under 80%H_2_ /20% CO_2_ atmosphere (1 bar) at 37°C and pH 7, as recommended by the Leibniz Institute DSMZ [10,57–60]. This medium was compared to a newly adopted culture medium called SAB medium to improve the culture and isolation of mesophilic methanogens associated with the human microbiota. *M. oralis* DSMZ 7256 grew faster in the SAB medium (three-day incubation with a 18-h doubling time) than in the modified DSMZ 119 medium (seven-day incubation and a 21-h doubling time) [58]. SAB medium was used to establish the repertoire of methanogens cultivated from severe periodontitis [2] and to study *M. oralis* genetic variants [55]. Subsequently, the SAB medium was optimised for bedside sampling, enabling the aerobic culture of methanogens including *M. oralis* [61]. Oral samples were collected in an Ae-Ana transport medium (Culture-Top, Marseille, France) initially designed for aerobic conservation during the sampling and transport of anaerobic bacteria. After this, 1 mL was aerobically transferred into a Hungate tube containing 5 mL of a modified liquid SAB medium with a growing culture of H_2_-CO_2,_ producing *Bacteroides thetaiotaomicron* (*B. thetaiotaomicron*). The composition of the medium was supplemented with three antioxidants (uric acid, ascorbic acid and glutathione), while glucose was required for *B. thetaiotaomicron* growth. After nine days, methane-positive cultures were inoculated onto SAB modified-solid plates with antibiotics to remove bacteria and fungi, and placed into the upper part of the two-chamber flask and incubated at 37°C for seven days. Ultimately, nine *M. oralis* strains were isolated. This technique was also used to isolate *M. oralis* from oral fluid [33,62] and human milk [34]. Furthermore, in order to routinise methanogen culture in the laboratory, hydrogen-producing *B. thetaiotaomicron* was successfully replaced by 1.5 g of iron filings, 200 mL of distilled water and 150 µL of acetic acid. The action of this weak acid on the iron produced sufficient hydrogen to enable the isolation of four additional strains of *M. oralis* [36].

Growing colonies could be identified by peptide profiling using matrix-assisted laser desorption/ionization time-of-flight mass spectrometry (MALDI-TOF-MS) as a rapid and low cost technique to identify cultured microorganisms including archaea, using one of the two reported protocols [57,62]. Both protocols involved a specific protein extraction from the broth medium. *M. oralis* reference spectra were absent from the Brüker-Daltonics database, but five *M. oralis*-strain spectra were added to our laboratory database, and successfully identified *M. oralis* from 14 clinical isolates [57,62].

### Antimicrobial susceptibility testing

*M. oralis* was initially isolated with MB medium containing cefalotin, clindamycin, kanamycin, and vancomycin, and several dilutions were necessary to obtain a pure culture [3]. Later, the antimicrobial resistance pattern of methanogens was determined using the macrodilution technique. *M. oralis* was found to be resistant to amphotericin B, ampicillin, streptomycin, gentamycin, rifampicin, ofloxacin, tetracycline (MIC > 100 mg/L), and vancomycin (MIC > 50 mg/L); moderately susceptible to chloramphenicol and bacitracin (MIC < 25 mg/L); and susceptible to metronidazole and ornidazole (MIC < 1 mg/L) [10]. The *M. oralis* susceptibility profile was, therefore, similar to *M. smithii* except for bacitracin, to which *M. smithii* was susceptible (MIC < 1 mg/L). Also, the *M. oralis* genome lacks the chloramphenicol-O-acetyltransferase gene, despite its moderate susceptibility to chloramphenicol. A further study pointed towards resistance to chloramphenicol (MIC = 50 mg/L) and ceftriaxone (MIC = 100 mg/L), two antibiotics commonly used to treat brain abscesses [13]. The correlation of the anti-archaeal activity of imidazole derivatives was confirmed with their hydrophobicity, in particular, with the aim of improving periodontal treatment [59]. Later, *M. oralis* susceptibility was extended to biocides. *M. oralis* was found to be susceptible to squalamine (MIC = 0.5 mg/L) and derivatives (from 0.5 to 5 mg/L), peracetic acid used to disinfect medical devices (MIC = 1.5 g/L), and chlorhexidine (MIC = 0.2 mg/L) [60]. The anti-cholesterol pro-drug lovastatin was also found to be effective on human methanogens including *M. oralis* (MIC = 4 mg/L) by interfering with isoprenyl synthesis and disrupting cell wall synthesis but had no known effect on intestinal bacteria and was already known to inhibit methanogenesis in livestock [63]. As for clinical relevance, an initial study found no significant difference between metronidazole combined with amoxicillin and mechanical treatment versus mechanical treatment alone in reducing the prevalence of archaea in individuals and periodontal sites, with both approaches providing a significant reduction [64]. Another study showed that adding metronidazole, with or without amoxicillin, to mechanical treatment was more effective than mechanical treatment alone at reducing archaeal load in periodontal disease [65]. More anecdotally, the possible inhibitory effect of *Neolamarckia cadamba* leaf extract on *M. oralis* was reported by studying its relative abundance in an in vitro fermentative digester by metagenomic analysis [66]. Finally, antibiotics targeting infection-causing bacteria may prove ineffective against *M. oralis* and methanogens, given their enzymes, metabolic pathways, cell walls, and membranes, which are distinct from bacteria, leading to potentially significant clinical consequences.

## *M.* *oralis* in the human microbiota

**Figure 3.**
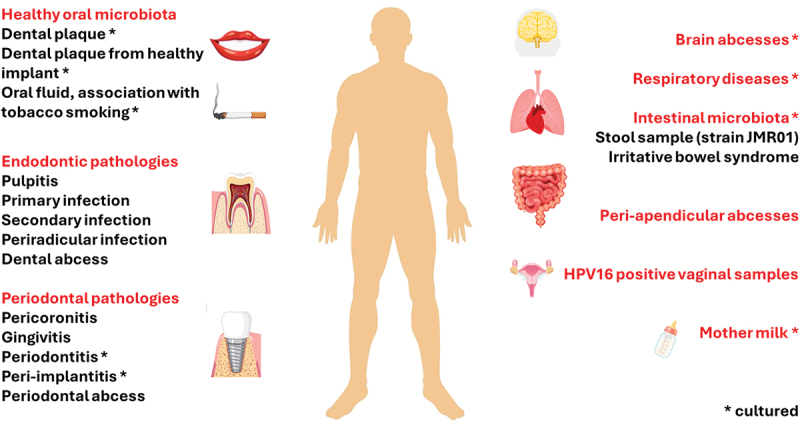
Localization and clinical insights of *M. oralis* in microbiota. The figure illustrates the presence of *M. oralis* in the human microbiota, depicting both oral and extra-oral locations and its association with dysbiosis or other pathological conditions. It highlights whether *M. oralis* has been cultured from associated clinical samples.

*M. oralis* was first isolated in the oral cavity, more specially from dental plaque samples of two healthy individuals, suggesting that it could be a member of the normal oral microbiota [3]. It also has been detected from the subgingival biofilm of healthy teeth and implants [28], and detected and cultured from the saliva of patients with no periodontal disease. However, a positive correlation was found between the occurrence of *M. oralis* and tobacco-smoking [33,36]. Moreover, *M. oralis* may occupy additional oral niches in pathological situations, including subgingival and pocket dental plaque from patients with periodontitis or peri-implantitis, pulp inflammation and infection (1). Finally, *M. oralis* appears to be the most prevalent methanogen associated with oral mucosa in both healthy and pathological situations, and also as a planktonic microorganism in the saliva. However, *M. oralis* does not seem to be restricted to the oral cavity, as it also has been detected in the human gut microbiota [46], the respiratory tract [37], and the vagina [49], and has been isolated from human faeces [52] and milk [34]. In particular, its adaptation to the human gut has not been clearly defined, as other studies did not report the presence of *M. oralis* [67], or reported it in a low prevalence [46,51,68]. A lack of correlation between the presence of methanogens in the gut and the oral cavity was mentioned by Brusa et al. in 1993 [69]. Given that *M. oralis* is unable to grow at a pH below 6, it may be destroyed by the acidity of the stomach [3]. Therefore, its colonisation within the human gut could occur in the presence of gastric pathologies or anti-acid treatments. Figure 3 illustrates the localization of *M. oralis* in human microbiota, in both oral and extra-oral locations.

### Acquisition and dynamics

The oral microbiota likely begins forming *in utero*, influenced by maternal microbiota and immunity, potentially contributing to a heritable component in shaping oral microbiota composition [70]. This early colonization primes infants for postnatal microbial exposures from their environment and interactions with others. Then, factors such as birth mode, feeding practices including breastfeeding, perinatal medications, teeth eruption, oral hygiene, sugar intake, antibiotics, maternal smoking, and the oral health of caregivers all significantly influence the development of infants’ oral microbiota [70]. These environmental influences persist throughout life and continue to shape the oral microbiota. However, the source of acquisition and the dynamics (*i.e*. changes and interactions within the oral microbiota over time, including colonization, growth, and response to environmental factors) of methanogens, particularly *M. oralis*, remain poorly studied.

Researchers have successfully cultured viable *M. oralis* from 1/20 (0.5%) maternal milk samples, suggesting a possible route of transmission through breastfeeding [34]. However, *M. oralis* was not found in the colostrum [34], meconium [71], or gastric juices of one-day-old newborns [53], in contrast to *M. smithii* [34,53,71]. Surprisingly, another study did not detect *M. smithii* but identified *M. oralis* as the predominant methanogen in meconium, placenta, and amniotic fluid samples from newborns, as well as in oral, rectal, and vaginal samples from mothers [54]. The use of archaeal-specific primers instead of bacterial-archaeal primers may have resulted in different identification of methanogen species. This study also indicates that neonatal microbial composition was not influenced by the delivery mode [54], contradicting findings from other studies [72]. Finally, researchers have not yet explored the presence of *M. oralis* in the oral cavity of newborns and older infants, leaving the key time when *M. oralis* is acquired unknown. Moreover, the easily exchangeable nature of oral fluids presents potential opportunities for the person-to-person transmission of *M. oralis*, occurring during interactions between mothers and children, including shared food consumption, fomite contact, and kissing [33]. This hypothesis gained support with the discovery of *M. oralis* in domestic baboons, which are in close contact with humans and share similar lifestyles and diets [23]. Moreover, the identification of *M. oralis* in the human-built environment hints at the possibility of environmental mediation in interhuman transmission [73]. In particular, multispacer sequence typing (MST) studying genetic variants of *M. oralis* may be useful in investigating the dynamics of *M. oralis* populations and inter-individual transmission [55]. Intriguingly, common foods such as confectionery products, fresh fruit, cheese, vegetables, meat, and fish do not appear to be a source of *M. oralis*, suggesting that food might not play a significant role in its transmission pathways [74]. However, diet and antimicrobials use can influence the microbiota throughout an individual’s lifetime, given that *M. oralis* thrives in symbiosis within specific bacterial niches. Dietary habits, particularly sugar intake, play a crucial role in shaping the oral microbiota [75]. High sugar consumption may promote the growth of certain bacteria that create an environment conducive to *M. oralis* proliferation, as evidenced by its notable rise since the industrial era [8,21,22]. Additionally, oral treatments such as mouthwashes can alter the microbial balance by selectively reducing or eliminating certain microbial populations, thereby impacting the overall composition of the microbiota [76]. *M. oralis*, for instance, is susceptible to chlorhexidine [60]. Antibiotic use, particularly the intake of metronidazole, can significantly influence the presence of *M. oralis*. Indeed, metronidazole is effective against anaerobic bacteria and can directly reduce *M. oralis* populations as it is susceptible to this antibiotic [10]. Conversely, other antibiotics might indirectly favor the growth of *M. oralis* by reducing competing bacterial populations, thus creating a niche where *M. oralis* can thrive. Therefore, external factors such as diet, oral hygiene practices, and antibiotic treatments may play a significant role in modulating the presence and abundance of *M. oralis* within the oral microbiota. However, these hypotheses should be confirmed by *in vitro* and clinical studies.

**Figure 4.**
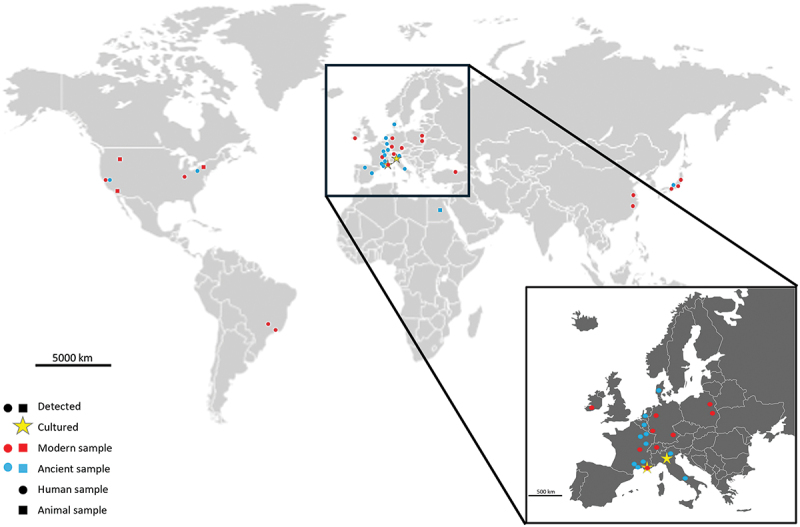
Geographical distribution and pathological contexts of *M. oralis* worldwide. The figure illustrates the global distribution of *M. oralis*, indicating its presence in various geographical locations and pathological contexts. In America, *M. oralis* has been found in sanitary indoor environments in the United States, as well as in animals such as baboons and cattle. In Europe, it has been cultured from mother milk, saliva, feces, and has been associated with conditions like periodontitis, peri-implantitis, and various respiratory diseases. Ancient dental calculus samples also revealed the presence of *M. oralis* in countries such as Belgium, France, Italy, and the Netherlands. In Asia, its presence is mainly noted in periodontitis, as well as in sanitary indoor environments in Japan and China. In Africa, traces of *M. oralis* have been discovered in ancient dental calculus in Egypt. *M. oralis* has been successfully cultured only in Marseille, France, and during its initial isolation in Milan, Italy.

Furthermore, *M. oralis* seems to exhibit a global distribution across multiple continents, including Europe, Africa, Asia, North, and South America (Figure 4). However, it is evident that not all countries and specific regions have been thoroughly investigated. Exploring diverse areas could prove valuable, particularly in studying the dynamics of transmission and its potential correlation with dietary patterns.

### *M. oralis* in pathological situations

#### Oral pathologies

##### Periodontal diseases

###### Gingivitis and periodontitis

Biofilm-induced gingivitis is a reversible inflammation limited to the gingiva, while periodontitis involves irreversible attachment loss, potentially leading to tooth loss [77]. Recent research on the microbiota of periodontal diseases has highlighted the importance of microbial dysbiosis, with shifts in the microbial community composition contributing significantly to the onset and progression of these diseases [78–80]. However, methanogens and *M. oralis* were not sufficiently considered in such studies.

Methanogens were firstly enriched from subgingival plaque in both healthy and periodontally diseased patients before the isolation of *M. oralis* from subgingival plaque samples collected from healthy individuals [3,25,26,69]. Only one study reported the detection of *M. oralis* in 2/3 (67%) Malian patients with gingivitis [65]. The first study detected methanogens by PCR-sequencing in 37/48 (77%) of patients with periodontitis, with no negative control group, and *M. oralis* was majoritarily identified (31/37; 84%) [27]. Despite the absence of a control group, this descriptive cross-sectional study is valuable as the first to identify *M. oralis* in periodontitis, highlighting the need for further research with control groups to validate and expand upon these findings. Later, a case control study with patients diagnosed with different stages of periodontitis and controls from healthy sites and healthy individuals was conducted, also with the aim of quantifying archaea and bacterial loads [4]. The prevalence of archaea was 36% for 50 periodontitis patients and *M. oralis* was identified in 81% of clones from six patients. Archaea appeared to be restricted to periodontitis sites, and a correlation between archaeal load and disease severity and bacterial load was established [4]. Importantly, no archaea were detected from healthy controls (sites or individuals), as confirmed in further studies [31,43,44,81]. The presence of methanogens was then associated with periodontitis-positive patients (11/49 (22%), 0/30 healthy patients) and pocket depth (>6 mm), and methanogens were detected in both chronic (6/32, 19%) and aggressive periodontitis (5/17, 29%) [31]. *M. oralis* and *M. oralis-*phylotype-like were predominant in four tested patients.

Additional studies have revealed the presence of *M. oralis* in healthy sites, yet consistently observed a higher prevalence and/or abundance in diseased sites, some with specific attention to chronic, aggressive, or mixed types of periodontitis. *M. oralis* was detected in 20 healthy individuals, and 20 patients diagnosed with aggressive periodontitis, but the abundance of archaea was significantly higher in diseased sites [82]. Moreover, the abundance of the archaeal species *M. oralis, M. smithii, Methanomassiliicoccus luminyensis*, and *M. stadtmanae* were significantly higher in periodontal disease when compared to healthy sites by metagenomics analysis. Functional analysis revealed that fermentation and methanogenesis were the predominant energy transfer metabolisms in disease [83]. The prevalence of *M. oralis* was significantly higher in chronic periodontitis patients (6/15, 40%) than in orally healthy individuals (1/15, 6.7%) [29]. Moreover, *M. oralis* was successfully identified in all seven sequenced samples, and a higher prevalence of methanogens was found in periodontitis sites (53% of mild periodontitis sites and 64% of moderate/advanced periodontitis sites) compared to peri-implantitis sites (10%) [84].

Accordingly, an *M. oralis*-specific RT-PCR system disclosed that *M. oralis* load significatively correlated with the periodontitis severity score, despite an absence of significant prevalence between periodontitis (12/22, 55%) and healthy patients (3/10, 30%) [9]. This correlation of *M. oralis* load with pocket depth (and age) was later confirmed, while the correlation with gender was not [40]. Finally, Huynh et al. were the only team to reintroduce the methanogen culture to confirm the presence of living microorganisms [2]. Their results showed that *M. oralis* was present in a living state in 31/65 patients (47.7%) with periodontitis, compared to only 1/15 healthy controls (6.7%), suggesting that studies based solely on PCR may overestimate the presence of viable *M. oralis* in healthy individuals’ samples, providing further evidence that *M. oralis* is implicated in periodontitis [2].

The role of *M. oralis* in periodontal diseases remains ambiguous, raising the question of whether it is a true pathogen or merely an opportunistic presence. Studies show a higher prevalence of *M. oralis* in periodontitis patients compared to healthy individuals, but its presence in healthy sites suggests a more complex relationship. The correlation between *M. oralis* load and disease severity indicates potential pathogenic involvement, yet its detection predominantly in diseased individuals highlights the need for further research. Current case studies and cross-sectional case-control studies have limitations; future research should include larger sample sizes and more diverse study designs, such as longitudinal studies to track changes over time or interventional studies to assess the impact of targeted elimination of *M. oralis*. These approaches combined with *in vitro* experimentation will provide more comprehensive evidence to determine the true role of *M. oralis* in periodontal diseases.

###### Peri-implantitis

Peri-implantitis is an inflammatory disease similar to periodontitis and leads to soft tissue and bone loss with the appearance of a pocket around the implant.

*M. oralis* is the majoritarily detected methanogen in peri-implantitis sites (90% of clones), and a significantly higher abundance of archaea (12/25 (48%) of peri-implantitis sites) than in healthy implant sites in individuals with peri-implantitis (4/25, 8%) and in healthy individuals (2/25, 4%) has been reported [28]. However, the methanogens association with peri-implantitis remains controversial, as they have been detected in both peri-implantitis sites (15/30, 50%) and healthy sites (16/28, 57%) without any significant difference [47]. However, a recent study detected no methanogen in healthy sites versus in 10% of peri-implantitis sites, 53% in mild periodontitis sites, and 64% in moderate/advanced periodontitis sites [84]. In contrast, another metagenomics study, which did not identify methanogens at the species level, revealed that the *Methanobrevibacter* genus was more abundant in peri-implantitis than in periodontitis [85] and suggested that the core microbiota of individuals with peri-implantitis and periodontitis are different. This contradiction deserves more investigation. Finally, the association of *M. oralis* with peri-implantitis appears even less evident than with periodontitis, highlighting the need for further research to clarify its pathogenic or opportunistic role.

##### Pericoronitis

Pericoronitis is an infectious disease of the soft tissues around partially erupted teeth, especially third mandibular molars [86]. *M. oralis* was detected by the PCR-sequencing method in 3/11 samples (27%) of subgingival plaques of third molars with symptomatic pericoronitis and was not detected in the 7 asymptomatic molars and in 1/11 (9%) control incisors [32]. This single study seems to align with the growing trend suggesting that *M. oralis* is more strongly associated with periodontal pathologies. However, caution is warranted as it has also been found in a healthy periodontal sample, and the study is limited by its sample size. Further research with larger samples is needed to fully elucidate the role of *M. oralis* in pericoronitis.

##### Endodontic pathologies

The endodontic microbial community is mainly composed of anaerobes, is less diverse in the secondary infection [87], and archaea are not so often included in studies.

A first study erroneously concluded that there was no archaea implication in the endodontic infection after failing to detect archaeal DNA. This was probably due to use of the wrong primers [88]. An *M. oralis*-like species was finally detected by RT-PCR in 25% (5/20) asymptomatic primary infected dental pulp samples, representing 0.28% to 2.53% of the total microbial community [42]. *M. oralis* was formally identified shortly after in the same samples [44]. The presence of *M. oralis* was again reported as the main archaea in the primary infection (59.4% (19/32) [38,39] as well as in the secondary infection (37.5% (12/32), [41,89]), and in inflamed pulp (85% (17/20), [90]), underscoring its potential implication across different stages of endodontic infections. Furthermore, Vickerman et al. [30] did not manage to establish a link between the symptomatic status and the presence of *M. oralis* in the root canal, as an *M. oralis*-like species was detected in 1/20 (5%) asymptomatic patients and 1/14 (7%) symptomatic patients. However, another study reported a significantly higher number of symptomatic cases positive for both bacteria and archaea (16/22, 73%) compared to cases positive for bacteria alone (21/47, 45%), without identifying archaea at the species level [91]. Two studies using bacterial 16S rRNA PCR analysis reported the presence of *M. oralis*. In the first study, *M. oralis* was detected in the root apex and periradicular soft tissue in 1/16 (6.25%) samples [92]. In the second study, *M. oralis* was found in 1/6 (16.7%) teeth before endodontic treatment but not afterward, suggesting that the endodontic procedure effectively removed *M. oralis* from the infected canal [93].

These data suggest that *M. oralis* may be a neglected member of the pathological microbial community in the endodontic infection process. The association between infection progression and symptomology remains unclear and warrants further investigation. Moreover, the efficacy of endodontic treatments should be studied on larger and more diverse samples, considering different available procedures, to better understand the role of *M. oralis* in the treatment outcomes of endodontic infections.

#### Extra-oral pathologies

##### Abscesses

*M. oralis* has been associated with several cases of infection and abscesses, revealing its potential pathogenicity for other sites than periodontal or endodontic tissue. Indeed, *M. oralis* was detected in 1/11 (9%) brain abscesses, confirmed by metagenomic analysis [14]. *M. oralis* was then cultured from one index brain abscess pus specimen, and RTQ-PCR reported that *M. oralis* had a significantly higher prevalence in brain abscesses than in other brain tissue controls, being detected in 7/8 (87.5%) of methanogen-positive pus specimens (7/18, 39.8% of the total brain abscesses pus specimens), and in 1/27 (3.7%) controls without brain abscess [13]. *M. oralis* was also reported in 1/4 (25%) cases of peri-appendicular abscesses [94], always with other bacteria, and in 2/100 (2%) cases of orthopaedic prosthesis infection [95]. These observations prompt a re-evaluation of its potential dissemination through the bloodstream, despite the fact that it has not been detected in archaemia [35].

##### Respiratory diseases

*M. oralis* was detected in 5/12 (42%) pus specimens positive for methanogens (12/116, 10.3%) and cultured from one of them (1/5, 20%) in patients with refractory sinusitis who had not received nitroimidazole derivatives [96]. However, a cross-sectional case-control study revealed no association between archaea and chronic rhinosinusitis with archaea detected in 2/20 (10%) healthy controls and 5/40 (12.5%) in the disease groups [38]. Moreover, a prospective study detected *M. oralis* in the respiratory tract from 19/527 (3.6%) sputum samples and 1/188 (0.53%) bronchoalveolar lavages but not in the 193 bronchial aspirates [37]. Further studies are needed to elucidate the potential involvement of *M. oralis* and methanogens in respiratory diseases.

##### Inflammatory diseases

*M. oralis* was identified in pooled fecal samples of patients with Crohn’s Disease (29/48, 60% of the sequenced clones) and irritable bowel syndrome (3/48, 6.3% of the sequenced clones), but no correlation was found between *M. oralis* and digestive tract diseases. Methanogens were detected in equivalent numbers of individuals (range from 45% to 50%, mainly corresponding to *M. smithii*) in colorectal cancer, polypectomised, irritable bowel syndrome, and control groups. Their prevalence was notably reduced in the inflammatory bowel disease groups, with 24% for ulcerative colitis and 30% for Crohn’s disease [46].

##### Cancer

Less is known about methanogens and cancer, however one study [39] showed a depletion (decrease in the abundance) of methanogens in patients with colorectal cancer, while another [45] revealed that archaeal metabolites, especially in the oral cavity and gut, could have an influence on the tumour microenvironment and carcinogenesis. A further study reported a positive correlation between colorectal cancer and *M. smithii*, but no data was provided concerning *M. oralis* [97]. However, a LEfSe (Linear discriminant analysis Effect Size) analysis was conducted to identify microorganisms that significantly differed in abundance between the studied groups and revealed a significant contribution of *M. oralis* to the differentiation between HPV16-positive and HPV16-negative groups of women, suggesting a potential association between *M. oralis* and HPV16 infection [49]. Given the carcinogenic nature of HPV16, this association assumes heightened importance, emphasising the need for further exploration of the role of *M. oralis* in the composition of the vaginal microbiome and its potential implication in cancer predisposition among women infected with HPV16.

#### Questioning pathogenicity and host response

While aggressive periodontitis does not necessarily require antibiotics to reduce the prevalence of archaea [64], periodontal treatment consisting in root scaling with adjunctive antibiotics for patients with chronic periodontitis is more effective at reducing archaea and the prevalence and load of *M. oralis* than mechanical treatment alone [65]. Both treatments, however, are associated with an improvement in periodontal health [65]. In particular, this reduction was not influenced by changes in prokaryotic biomass. The same findings emerged concerning root canal treatment, where *M. oralis* was not detected after irrigation with 3% H_2_O_2_ and intracanal medication, and there was no significant difference in total DNA extracted before and after treatment, indicating a shift in the microbial species present rather than a significant reduction in the overall microbial load [93]. However, these results are insufficient to conclude that *M. oralis* plays a role in endodontic pathologies, as only six samples were included, and root canal treatment and irrigation solutions are not specifically targeted against *M. oralis*. It may simply be part of the complex microbiota and act opportunistically.

The first direct evidence of pathogenicity was provided by the inoculation of an *M. oralis* monoculture or *M. oralis* with *S. intermedius* in a mouse model leading to animal death (17/22 (77.3%) and 75/104 (72.1%) mice died, respectively). This was significantly higher than deaths caused by inoculation of *S. intermedius* alone (32/95 (33.7%), showing for the first time the direct pathogenicity of this methanogen [13]. Studies were then conducted focusing on the immunogenicity of *M. oralis*, revealing that sera from patients with periodontitis contained IgG against *M. oralis* [98]. These antibodies target *M. oralis* group II chaperonin (Cpn-1 and Cpn-2 subunits) and show potential cross-reactivity with human group II chaperonin CCT. This suggests their relevance in periodontal diseases and prompts further investigation into their potential involvement in autoimmune responses [98,99].

## *M.* *oralis* in animal microbiota

Few recent studies have reported the presence of *M. oralis* in animals’ microbiota. It was notably detected in 7/10 (70%) dogs with severe periodontitis but not in healthy dogs or those with mild or moderate periodontal disease, hinting at a potential connection to canine gum health [48]. Additionally, dental calculus analysis identified *M. oralis* in a 19^th^-century baboon and a mummified domestic baboon from the Pharaonic era (9^th^–6^th^ centuries BP). This discovery not only provides insights into the ancient oral microbiota of animals but also raises questions about potential human influences on the potential interspecies transmission of *M. oralis*. Interestingly, a contemporary wild chimpanzee, in contrast, showed no evidence of *M. oralis*, underscoring the distinctive variations in oral microbial communities among closely related primate species [23]. Likewise, while some studies reported the presence of *M. oralis* in the rumen of non-dairy and dairy cows [100,101], a comprehensive study by Guindo et al. did not confirm these observations when it investigated dogs, cats, cows, sheep, horses, and pigs, in which *M. oralis* was not detected [102]. Finally, *M. oralis* has been found in only three animal species: baboons, dogs, and cows [23,48,100,101]. However, this also may arise from the limited number of samples included and the focus on gut or faecal samples in animal studies, while samples from the oral cavity are not routinely examined.

## *M.* *oralis* and other microorganisms

### *M. oralis* and bacteria

**Figure 5.**
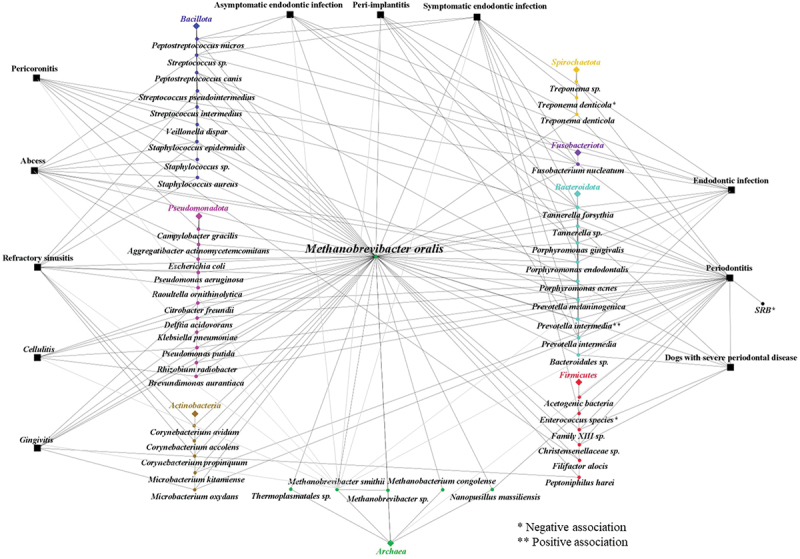
Interactions of *M*. oralis with various bacteria and archaea in different pathological conditions. This figure illustrates the complex network of interactions between *M. oralis* and various bacterial and archaeal species across different pathological conditions. The connections highlight both positive and negative associations with other microbes, indicating potential synergistic or antagonistic relationships.

*M. oralis* has been identified alongside various previously selected and targeted bacteria in different oral pathologies, suggesting potential symbiotic partnerships (Figure 5). In cases of pericoronitis, *M. oralis* was consistently present in all sites which were positive for *Fusobacterium nucleatum* (*F. nucleatum*), while *Campylobacter gracilis*, *Prevotella melaninogenica*, *Veillonella dispar, Filifactor alocis*, and *Tannerella forsythia* (*T. forsythia*) were found in both positive and negative archaeal sites [32]. Furthermore, in one symptomatic endodontic case, *M. oralis* was co-detected with *Prevotella intermedia* (*P. intermedia*), *Porphyromonas endodontalis* (*P. endodontalis*), *Porphyromonas gingivalis* (*P. gingivalis*), *Peptostreptococcus micros* (*P. micros*), *Streptococcus* sp., *F. nucleatum*, and *T. forsythia*, but not with *Treponema denticola* (*T. denticola*) or *Enterococcus* species, while in an asymptomatic case, it was detected alongside *P. gingivalis, P. micros, Streptococcus sp., F. nucleatum*, and *T. forsythia* [30]. Interestingly, no specific associations were reported with the selected bacteria (*T. denticola, T. forsythiae, P. gingivalis, F. nucleatum, P. intermedia*) in cases of periodontitis and peri-implantitis [84]. Furthermore, despite the formation of a unique core microbiome in severe periodontal disease cases in dogs, including *M. oralis*, *Christensenellaceae sp, Bacteroidales sp*, Family XIII sp, *Peptostreptococcus canis*, and *Tannerella* sp, the correlation of *M. oralis* with bacterial species has not been studied [48]. Other studies have specifically reported both positive and negative associations with *M. oralis*, contributing to a better understanding of complex microbial relationships. In periodontitis, a negative association was found with *Treponema* spp. [4], despite the co-detection of *T. denticola* with *M. oralis* in an asymptomatic endodontic sample and its absence in another *M. oralis*-positive symptomatic sample [30]. The negative association of archaea, including *M. oralis*, with *T. denticola* was confirmed in another study, possibly explained by their shared role as hydrogen consumers [41]. Similarly, mutual exclusion with other hydrogen consumers such as sulfate-reducing bacteria (SRB) and acetogens suggests their potential as alternative syntrophic partners for secondary fermenting periodontal pathogens [43]. Horz et al. supported the positive association with *P. intermedia* in endodontic infections, noting that *P. intermedia* has a broader substrate range for fermentation, including carbohydrates and proteins, compared to the asaccharolytic *Porphyromonas* and *Tannerella* within the *Bacteroides* phylum, which may be more suitable for *M. oralis* [40]. Interestingly, in Malian patients with different oral conditions, such as gingivitis and periodontitis, *M. oralis* was detected with bacteria that were not reported in other studies including *Delftia acidovorans, Microbacterium oxydans, Pseudomonas putida*, *Citrobacter freundii, Brevundimonas aurantiaca, Rhizobium radiobacter, Microbacterium kitamiense, Peptoniphilus harei* and *Klebsiella pneumoniae*. This finding suggests that the microbial partnerships of *M. oralis* may vary depending on the geographic location, shedding light on potential regional differences in oral microbiota composition [65].

Moreover, *M. oralis* has been found alongside other bacteria in diverse extra-oral conditions (Figure 5). These bacteria were detected and cultured impartially, without prior biases, to explore the microbial diversity of the sample. *M. oralis* has been identified in cases of refractory sinusitis, coexisting with various bacterial species, including *Corynebacterium accolens*, *Staphylococcus aureus* (*S. aureus*), *Raoultella ornithinolytica*, *Streptococcus pseudintermedius*, *Pseudomonas aeruginosa* (*P. aeruginosa*), *Staphylococcus epidermidis* (*S. epidermidis*), *Corynebacterium propinquum*, *Corynebacterium avidum*, and *Propionibacterium acnes* [96]. In particular, *S. epidermidis* was found in all four cases positive for *M. oralis*, *P. aeruginosa* in two out of four cases, and *Corynebacteria* species in three out of four cases. Furthermore, *M. oralis* was identified in a peri-appendicular abscess with *Escherichia coli* [94], in a brain abscess with *Aggregatibacter actinomycetemcomitans* [14], and in another case of a brain abscess with *Porphyromonas endodontalis* and with *S. intermedius* [13]. Co-infection experiments in mice revealed significantly higher mortality rates when *M. oralis* was present with *S. intermedius*, indicating the severity of the combined infections [13]. Additional co-detection with *S. aureus* and *S. epidermidis* in orthopaedic prosthesis infection cases suggests a potential symbiotic relationship with *Staphylococcus* sp. [95]. Given the facultative anaerobic nature of these two species, enabling adaptation to both aerobic and anaerobic conditions, coupled with their inherent defence mechanisms against oxygen and reactive oxygen species (ROS), the establishment of a biofilm may provide a favourable niche for methanogens to thrive [95].

While methanogens, including *M. oralis*, have traditionally been viewed as secondary colonisers, relying on syntrophic interactions with bacterial partners, particularly in hydrogen transfer, their mere co-detection with bacteria does not necessarily imply dependency, and most of the studies presented here focused only on known oral pathogens. Investigating positive associations may reveal potential partners for *M. oralis* such as *P. intermedia*. Such insights hold the potential to enhance cultivation techniques. Conversely, exploring negative associations, such as competition, particularly in terms of hydrogen utilisation and varying degrees of mutual exclusion, notably with *Treponema* sp., may be useful for a comprehensive understanding of microbial community dynamics. Further research, incorporating correlation analysis and co-culturing, is pivotal to produce nuanced insights into both dependencies and competition among microorganisms.

### *M. oralis* and other archaea

- *M. oralis* was the predominant methanogen in oral samples and was mainly detected alone, suggesting competition within the *Methanobrevibacter* genus. However, some studies revealed that this co-exclusion was not strict and *M. oralis* seemed to be able to coexist with other *Methanobrevibacter* (Figure 5). Indeed, *M. oralis* was co-detected in 3/34 subgingival dental plaque samples, 31 of which contained only one phylotype [27]. Moreover, *M. oralis* was co-detected with *Methanobrevibacter* phylotype SBGA-1 in four plaque samples from four patients with periodontitis [31] and was also co-detected with *Methanobacterium congolense/curvatum*. This is a hydrogenotrophic methanogen initially isolated from an anaerobic digester in Congo, in a peri-implantitis group and in a healthy control group, although the other methanogen had a lower prevalence [28]. *M. oralis* was co-detected in endodontic samples with a *Methanobrevibacter* species associated with *Synergistes* sp [44]. However, the ability of *M. oralis* to share its ecological niche was confirmed by Grine et al. who co-cultured *M. oralis* and *M. smithii* from saliva [36].
- *M. oralis* has also been detected alongside other members of the archaea domain (Figure 5). Firstly, *M. oralis* and a *Thermoplasmatales* species were co-detected in the oral cavity [103]. Then, *Nanopussillus massiliense* (*N. massiliense*) was co-detected with *M. oralis* in 4/102 (3.92%) dental plaque specimens and co-isolated with *M. oralis* from one dental plaque specimen [12]. *N. massiliense* was the first nanoarchaea isolated in the human microbiota. Nanoarchaea revealed a small genome with reduced metabolic function which appeared to be strongly dependent on their host. This finding added a new layer to our understanding of symbiotic relationships in microbial ecosystems. While methanogens were historically regarded as organisms which were heavily reliant on bacteria and their metabolic byproducts, this discovery provided a more nuanced perspective. It revealed that methanogens such as *M. oralis*, could also play a vital role as hosts, supporting the existence of smaller microorganisms.

## Conclusion and perspectives

*M. oralis* predominantly inhabits the human oral cavity, dating back to the Palaeolithic era and Neanderthal times [5,7], with its prevalence possibly influenced by societal changes like sugar consumption from the 18th century onwards [8,21,22]. However, our understanding of *M. oralis* is hindered by limited microbiological data, including a small number of genomes and cultured representatives [12,34,52,104]. Important phenotypic characteristics such as cell wall composition and metabolic pathways remain largely unexplored. Research into its interactions within specific oral microbiota niches, including symbiotic relationships with bacteria like *P. intermedia* [40], may clarify its predominance in the oral cavity and its potential role in various oral pathologies, particularly periodontitis. This point remains unresolved, as data are contradictory; whether *M. oralis* participates in dysbiosis, exacerbates it, or acts as a triggering factor is yet to be fully understood. Moreover, despite its presence in brain abscesses and other extra-oral locations, its systemic implications, especially concerning bloodstream dissemination [13,14,45], require further investigation. This could signify a broader impact on human health than oral pathologies, potentially supporting the development of other infections. If its pathogenic role is confirmed in the future, this could impact patient treatment, particularly due to its specific antibiotic resistance [10]. A positive note is that there are already limited but validated methods available to detect and quantify *M. oralis* in clinical situations. Finally, this review underscores the limitations in our current knowledge of *M. oralis* and emphasizes the necessity of integrating research on methanogens, particularly *M. oralis*, into both oral and broader general health studies.

## Data Availability

Data sharing is not applicable to this article as no new data were created or analysed in this study.
